# Age-Dependent Changes in Thermo–Viscoelastic Properties of Human Brain by Non-Equilibrium Thermodynamics with Internal Variables

**DOI:** 10.3390/biology15010070

**Published:** 2025-12-30

**Authors:** Annamaria Russo, Ester Tellone, Caterina Farsaci, Francesco Farsaci

**Affiliations:** Department of Chemical, Biological, Pharmaceutical and Environmental Sciences, University of Messina, Viale Ferdinando Stagno d’Alcontres 31, 98166 Messina, Italy; etellone@unime.it (E.T.); katefarsaci91@virgilio.it (C.F.); farsaci@ipcf.cnr.it (F.F.)

**Keywords:** brain, rheology, neurodegeneration, aging, thermodynamic, oxidative stress, non-equilibrium, thermo–viscoelastic

## Abstract

Over the years, neurons undergo functional changes initially linked to the maturation of the brain and then are progressively linked to normal aging. The curious relationship between brain decay, aging, and neuronal diseases has aroused the interest of numerous studies to better understand and contrast the evolution of these pathologies. The objective of this research is to apply the non-equilibrium thermodynamic theory with the internal variables of the study of the rheological properties of the brain, focusing on the study of viscoelastic properties. After a thermodynamic introduction of the principal rheological phenomena, this paper discusses the results by the application of our mathematical technique, which revealed a prevalence of anelastic properties in the old central nervous system compared to the young one. Furthermore, the entropy production trend tested identifies a greater disorder in the young brain in respect to the old one. The results obtained highlight that a lower stiffness in the old central nervous system may be interpreted with dendritic regression associated with neuronal death, both being potential consequences of an increased production of free radicals due to reduced antioxidant defenses and/or an altered mitochondrial dysfunction in aging.

## Simple Summary

The alterations to which neurons are subjected during their lifetime cause a general decline in cognitive activities as the years pass. The purpose of this research is to analyze the viscoelastic properties of the human brain using new mathematical procedures to allow for a thermodynamic characterization of this tissue. From this study, it is evident that the young brain is more rigid, less fluid, and more viscous than the old brain, and this is explained by remembering that the old one is characterized by neuron degeneration with partial myelin loss and a loss of “compactness”. Furthermore, in the brain, the oxidation of glucose for energy purposes is associated with the production of entropy, so the lower degree of entropy production monitored in the old brain compared to the young one inevitably indicates a lower metabolic activity of the neurons. In conclusion, this study of the rheological properties of the central nervous system through a thermodynamic approach has led to new characteristics of the young and old brain, allowing for new knowledge of the phenomena involved.

## 1. Introduction

The central nervous system (CNS), consisting of the brain and spinal cord, is the most complex of all biological organs. The brain includes the cerebrum (which controls thought, memory, voluntary movement), cerebellum (which regulates balance, coordination), brainstem (which coordinates basic life functions like breathing and heartbeat), and subcortical structures (which coordinate emotion, motivation); the spinal cord acts as a communication highway between the brain and the rest of the body, transmitting sensory input and motor commands.

CNS processing information, controlling movement, sensation, thought, and emotion, is essential for every aspect of human life, and its study not only advances medicine but also fuels breakthroughs in psychology, technology, and education.

In medicine, it is the foundation of neurology and neurosurgery, disciplines dedicated to diagnosing and treating disorders that affect the brain and spinal cord, such as epilepsy, stroke or Parkinson’s disease. Neuropharmacology also relies on this knowledge to develop drugs that act on neurotransmitters, offering new therapeutic options for conditions like depression, anxiety, and neurodegenerative diseases. Rehabilitation, too, benefits from the study of the CNS, since the principle of neuroplasticity makes it possible to design therapies that help patients recover functions after brain injuries or spinal cord trauma.

Psychology and psychiatry use insights into the CNS to better understand mental health conditions such as schizophrenia and mood disorders. They also apply this knowledge in therapeutic approaches, including cognitive–behavioral therapies, which exploit the mechanisms of brain functioning to improve emotional regulation and behavioral responses.

In the realm of technology and engineering, its applications are equally groundbreaking. Brain–computer interfaces allow paralyzed individuals to control external devices using their brain signals, restoring autonomy and communication. Artificial intelligence and robotics take inspiration from neural networks in the brain to create increasingly sophisticated systems capable of learning and adapting. Neuroprosthetics, meanwhile, enable the development of artificial limbs that respond directly to neural activity, integrating seamlessly with the body and enhancing patients’ independence.

Education and cognitive enhancement also benefit from CNS research. Studies on learning, memory, and attention help refine teaching methods and lead to the creation of cognitive training programs designed to slow age-related decline and promote effective lifelong learning.

In summary, the study of the central nervous system goes far beyond understanding how the human body works. It translates into practical applications that range from treating neurological diseases to creating advanced technologies and improving cognitive abilities. It is a field where science, health, and innovation converge, with profound and lasting impacts on society.

All this makes understanding the brain’s biochemical processes one of the most intriguing scientific pursuits. The CNS is mainly characterized by two types of cells immersed in the cerebrospinal fluid: neurons (excitable) and glial cells (not excitable). Cerebrospinal fluid has a composition largely similar to that of blood plasma, except for the protein content, which is much lower. This fluid assists the brain by providing protection and nourishment. Each neuron is generally in contact with thousands of synaptic terminals from which it receives information and transmits output signals in the form of action potentials via a single axon [1,2]. Over the years, neurons undergo important morphological and functional changes initially (up to about 20 years) linked to the maturation of the brain, and then progressively linked to the deterioration of normal aging [3]. These changes, affecting the cellular and subcellular structures and the numerous neuronal interconnections, characterize the entire organ and cause a general decline in cognitive activities. Sometimes, this compromise can also affect genetic factors and become well localized so as to give rise to the most common neurodegenerative diseases, including Alzheimer’s, Parkinson’s, Huntington’s and Amyotrophic Lateral Sclerosis [4,5,6]. The greatest damage to cellular structures is due to reactive oxygen species (ROS), also in consideration of the daily high oxygen demand of the brain. Curiously, both neurological changes due to age and pathologies deriving from the main neurodegenerative diseases ultimately involve oxidative stress produced during energy metabolism [7,8]. In reality, alterations of normal mitochondrial functions seem to be the main causes of neurological damage, and in this context, a clear dividing line between the damage caused by neurodegenerative diseases and aging is also not clearly deducible. For this reason, studying the changes in the brain caused by aging can also help to better understand the evolution of neurodegenerative diseases.

Significant advances have been made in the non-invasive field of neuroimaging technology, which has provided a deeper understanding of brain development. These techniques include magnetic resonance imaging, one of the most important neuroimaging techniques applied to the “in vivo” study of brain structures that can be used to evaluate changes in volume or thickness of specific structures over time; functional magnetic resonance imaging, an imaging method that demonstrates regional and time-varying changes in brain metabolism; positron emission tomography, which relies on the short half-life properties of radionuclides that emit positrons to map brain systems; and electroencephalography (EEG) and magneto–encephalography (MEG), two techniques based on ion currents caused by the exchange of information from neurons. EEG detects the electrical activity of active neurons using electrodes attached to the scalp, and MEG detects the oscillations of the magnetic field caused by these electrical currents. Near-infrared spectroscopy exploits the ability of light in the near infrared (650–900 nm) to penetrate biological tissues. The great diversity of these techniques means that each of them is a potential tool for studying a particular feature of the brain. In this paper, we propose an alternative technique for the study of the brain’s structure that makes use of the principles of rheology, a branch of physics that studies the deformation and flow of matter [9]. It is widely used to study the viscoelastic properties of soft biological materials, such as in the diagnosis of liver and breast tumors, because, by measuring parameters such as viscosity, elastic modulus and relaxation, the viscoelastic properties of a material characterize its ability to combine viscous (fluid) and elastic (solid) behavior when subjected to deformation or stress. These properties are crucial for study of body tissues as long as it is very difficult (if not impossible) to carry out experimental measurements on soft tissues, because there are great difficulties in the mechanical coupling between the sample and the measuring instrument. This occurs because these quantities are mechanical (stress) and kinematic (deformation) in nature. The study becomes significantly complicated when dynamic measurements are performed by subjecting the sample to a harmonic shear. However, complementary techniques have been developed that allow us to provide information on the quantities that characterize mechanical relaxation phenomena. One of these techniques, using elastic waves (ultrasound) and the relaxation described above, can be characterized by the so-called dynamic modules G1 and G2 by measuring the speed and attenuation of these waves (as a function of frequency) [10]. Another technique that can provide information of this type is multifrequency magnetic resonance elastography (MRE) [11].

The purpose of this study is to use of the MRE technique for the analysis of the viscoelastic properties of the brain with the application of a new mathematical approach of non-equilibrium thermodynamics. To this end, a complex mathematical reworking of the measurements made by Sack et al. was carried out that allowed for the thermodynamic characterization of the brain [11].

## 2. Materials and Methods

From a thermodynamic point of view, the brain and, in general, all living matter are out of equilibrium, and this is the reason why the characterization of our study applies the application of non-equilibrium thermodynamics. We used the thermodynamic approach with internal variables formulated by De Groot, Mazur, and Kluitenberg [12,13,14,15,16,17], which was developed and deepened in subsequent studies [18], for the thermodynamic quantities calculation of the theory using experimental data. This was possible because we determined the interconnected relationships between the thermodynamic functions of the theory and the quantities that are experimentally measured in both the mechanical [10,18] and electrodynamic [19] cases. The data used in this paper are derived from multifrequency magnetic resonance elastography measurements on healthy human brains of 55 volunteers (31 males, age range 21 to 84 years; 24 females, age range 18 to 88 years) [11]. In detail, the application of four vibration frequencies in an acoustic range from 25 to 62.5 Hz and the use of the rheological spring model allowed for the determination of two parameters describing the solid–fluid behavior and microstructure of the brain tissue [11].

### 2.1. Non-Equilibrium Thermodynamic Approach with Internal Variables

The basic concepts of Kluitenberg’s theory and the subsequent developments are recalled here [12,13,14,15,16,17,18,19]. We will make extensive use of concepts regarding elastic and anelastic phenomena related to the respective deformations; for this reason, we specify below what is meant by elastic and anelastic deformations [20].

(1) Elastic deformation is that part of the deformation that is instantly recovered; therefore, it is a reversible process (it does not appear in the production of entropy).
(2) Anelastic deformation is the deforming part recovered after a finite time; therefore, with finite speed. According to Planck, it is irreversible and dissipative (it appears in the production of entropy). In the real world, elastic deformation does not exist—it is approximated.

### 2.2. Theoretical Thermodynamic Approach

Kluitenberg’s theory is based on the idea that the usual variables of non-equilibrium thermodynamic are insufficient to describe some phenomena that occur in a medium when it is subject to perturbation. In particular, they are insufficient to describe relaxation dielectric phenomena in a continuous media (we neglect the magnetic effects).

Generally, it is assumed that the specific entropy *s* of an elastic dielectric is a function of the specific internal energy u and the strain tensor *ε_ik_*:

(1) $$s=s\left(u\left|\varepsilon_{ik}\right.\right)$$

The new Kluitenberg idea consists of the assumption that there is a vector field $\varepsilon_{ik}^{\left(1\right)}$ that plays the role of the thermodynamic internal degree of freedom, which influences the strain. In the theory, it is assumed that the specific entropy (which we indicate with s) has the following functional dependence [17]:

(2) $$s=s\left(u\left|\varepsilon_{ik}\left|\varepsilon_{ik}^{\left(1\right)}\right.\right.\right)$$

The tensor strain is additively composed of two parts $\varepsilon_{ik}^{\left(0\right)}$ and $\varepsilon_{ik}^{\left(1\right)}$ [14]:

(3) $$\varepsilon_{ik}=\;\varepsilon_{ik}^{\left(0\right)}+\;\varepsilon_{ik}^{\left(1\right)}$$

Moreover, it can be shown that the change of both $\varepsilon_{ik}^{\left(0\right)}$ and $\varepsilon_{ik}^{\left(1\right)}$ contributes to entropy production, and therefore, they represent two irreversible processes.

So, we can introduce the viscous stress tensor $\tau_{ik}^{\left(vi\right)}$:

(4) $$\tau_{ik}^{\left(vi\right)}=\;\tau_{ik}-\;\tau_{ik}^{\left(eq\right)}$$

Here, $\tau_{ik}$ is the stress tensor that occurs in indefinite equations. If the irreversible field (4) vanishes, the change in $\varepsilon_{ik}^{\left(0\right)}$ does not contribute to the entropy production, i.e., changes in $\varepsilon_{ik}^{\left(0\right)}$ are reversible processes.

### 2.3. Phenomenological Equations

From Equation (2), we obtain:

(5) $$\frac{ds}{dt}=\;\frac{\partial s}{\partial U}\frac{dU}{dt}+\;\frac{\partial s}{\partial {Ɛ}_{ik}}\frac{d{Ɛ}_{ik}}{dt}+\;\frac{\partial s}{\partial {Ɛ}_{ik}^{\left(1\right)}}\;\frac{d{Ɛ}_{ik}^{\left(1\right)}}{dt}$$

The entropy production per unit of volume and per unit of time is given by [17]:

(6) $$\begin{array}{l} \sigma^{\left(s\right)}=\frac{1}{T}\left[\tau_{ik}^{\left(vi\right)}\frac{d\varepsilon_{ik}}{dt}+\tau_{ik}^{\left(1\right)}\frac{d\varepsilon_{ik}^{\left(1\right)}}{dt}\right]=\\ =\frac{1}{T}\left[\tau_{ik}^{\left(vi\right)}\frac{d\varepsilon_{ik}^{\left(0\right)}}{dt}+\left(\tau_{ik}^{\left(vi\right)}+\tau_{ik}^{\left(1\right)}\right)\frac{d\varepsilon_{ik}^{\left(1\right)}}{dt}\right] \end{array}$$

In agreement with the method of non-equilibrium thermodynamics developed by De Groot and Mazur, there will be linear relations among these quantities, which, for an isotropic media, can be written as [12,13,14,15,16,17,18]:

(7) $$\tau^{\left(vi\right)}=\eta_{s}^{\left(0,0\right)}\frac{d\varepsilon }{dt}+\eta_{s}^{\left(0,1\right)}\tau^{\left(1\right)}$$

(8) $$\frac{d\varepsilon^{\left(1\right)}}{dt}=\eta_{s}^{\left(1,0\right)}\frac{d\varepsilon }{dt}+\eta_{s}^{\left(1,1\right)}\tau^{\left(1\right)}$$

where $\eta_{s}^{\left(0,1\right)},\eta_{s}^{\left(1,0\right)},\eta_{s}^{\left(0,0\right)},\eta_{s}^{\left(1,1\right)}$ are phenomenological coefficients, and we assume that they are constant over time. The coefficients $\eta_{s}^{\left(0,1\right)},\eta_{s}^{\left(1,0\right)}$ are connected with possible cross effects that may occur between the two types of mechanical relaxation phenomena described by Equations (7) and (8), and they satisfy the Onsager–Casimir reciprocal relations:

$$\eta_{s}^{\left(0,1\right)}=-\eta_{s}^{\left(1,0\right)}$$

If we neglect the cross effect described by coefficients ($\eta_{s}^{\left(0,1\right)},\eta_{s}^{\left(1,0\right)}$), we observe that the coefficient $\eta_{s}^{\left(0,0\right)}$, which has the dimension of a viscosity, is connected to irreversible processes related to the change of Ɛ, while $\eta_{s}^{\left(1,1\right)}$, which has the dimension of a fluidity, is related to change of $\varepsilon^{\left(1\right)}$ and the corresponding intensive variable $\tau^{\left(1\right)}$. However, Equations (7) and (8) are connected with irreversible changes of the strain [15].

For a tissue like the one we are studying in this work, it is reasonable to assume that ρ is constant for each element in order to verify the fundamental axioms on local and instantaneous equilibrium.

### 2.4. Linear Response Theory

In this paper, the mechanical relaxation phenomena will be studied, so the medium under examination is assumed as being subjected to harmonic stress. In this context, the perturbation is an extensive quantity (cause), and the corresponding intensive quantity (effect) will be studied. It will be assumed that:

ε = ε_o_ senωt (9)

extensive variable (cause).

And thus it will be [21,22]:

(10) $$\tau =\tau_{o}sen(\omega t+\delta )$$

intensive variable (effect) where δ is a phase lag.

We do not go into the details of Equations (9) and (10) that are part of the linear response theory. We simply want to introduce the complex dynamic module G = G_1_ + iG_2_.

From Equation (10), it is:

(11) $$\tau =\tau_{o}sen\omega tcos\delta +\tau_{o}cos\omega tsen\delta$$

(12) $$\tau =\varepsilon_{o\;}\;\left(\frac{\tau_{o}}{\varepsilon_{o}}cos\right)sen\omega t+\varepsilon_{o\;}\left(\frac{\tau_{o}}{\varepsilon_{o}}sen\delta \right)cos\omega t$$

Placing:

(13) $$G_{1}=\frac{\tau_{o}}{\varepsilon_{o}}\;cos\delta$$

(14) $$G_{2}=\frac{\tau_{o}}{\varepsilon_{o}}\;sen\delta$$

Equation (12) is written [21]:

(15) $$\tau ={(\varepsilon }_{o\;}G_{1\;})sen\omega t+{(\varepsilon }_{o\;}G_{2\;})cos\omega t$$

G_1_ and G_2_, called storage and the loss modulus, are related to elastic and dissipative phenomena, respectively.

### 2.5. State and Phenomenological Coefficients (Explicit Form)

The medium studied is supposed to undergo a shear deformation of the type:

$$\varepsilon =\varepsilon_{o\;}sen\omega t$$

The following relationship between phenomenological and state coefficients and G_1_ and G_2_ can be shown [18]:

(16) $${\;a}^{\left(0,0\right)}=G_{1\;}+\frac{G_{2}^{\left(1\right)}}{\omega \sigma }$$

(17) $$a^{\left(1,1\right)}=\frac{{(G}_{1}\omega \sigma +G_{2}^{\left(1\right)})2}{\omega \sigma (G_{2}^{\left(1\right)})(1+\omega^{2}\sigma^{2})}$$

(18) $$\eta_{s}^{\left(1,1\right)}=\frac{(G_{2}^{\left(1\right)})(1+\omega^{2}\sigma^{2})\omega \;}{(G_{1}\omega \sigma +G_{2}^{\left(1\right)})2}$$

To complete the system, the following equation is introduced [19]:

(19) $$\eta_{s}^{\left(0,0\right)}=\frac{G_{2R}}{\omega }$$

where *G*_2*R*_ is the relaxed value of G_2_ [18]. We justify Equation (19). The viscous phenomenon associated with $\eta_{s}^{\left(0,0\right)}$ is a dissipative phenomenon, and therefore, it must be somehow “contained” in G_2_, representing all the dissipative phenomena, both viscous and anelastic. Now, if G_2_ represents only viscous phenomena, it is known that $\eta_{s}^{\left(0,0\right)}$ = $\frac{G_{2}}{\omega }$. But it is known that the viscous phenomena are more evident at low frequencies where G_2_ varies little, and therefore, it seems reasonable to approximate $\eta_{s}^{\left(0,0\right)}$ = $\frac{G_{2}}{\omega }$ with Equation (19). From Equation (19), the term follows:

$$G_{2}^{\left(1\right)}=G_{2}-\eta_{s}^{\left(0,0\right)}\omega =G_{2}{-G}_{2R}$$

which can be considered the inelastic “part” of the loss modulus G_2_ as the viscous part is subtracted. Module $G_{2}^{\left(1\right)}$ will be called the anelastic loss modulus. Equations (16)–(19) give the four coefficients as a function of the perturbation frequency ω. It is easy to see that they are positive.

### 2.6. Relaxation Equation

In the following, we assume that T = constant, in agreement with physiological phenomena. The importance of the phenomenological and state coefficients is that they characterize the medium specifying the amount of the type of phenomena correlated to each of them.

It is important to observe that their constancy refers to the time for each type of perturbation that acts on the medium. But they vary with the change of the perturbation. For example, if the perturbation is of harmonic type with frequency ω, then the coefficients will depend on ω (we will see this in the next section), which can be considered the parameter in the functional dependence of the coefficients. In this case, we will call $a^{\left(0,0\right)},a^{\left(1,1\right)},\eta^{\left(0,0\right)},\eta^{\left(1,1\right)}$ *dynamical coefficients*.

### 2.7. Thermodynamic Functions

Considering the thermodynamic functions introduced above, we can define a matrix that we will yield “Thermodynamic Matrix Dynamic Behavior” (TMDB):

$$TMDB=\left\|\begin{array}{cccc} a^{\left(0,0\right)} & a^{\left(1,1\right)} & \eta_{s}^{\left(0,0\right)} & \eta_{s}^{\left(1,1\right)}\\ \varepsilon^{\left(0\right)} & \varepsilon^{\left(1\right)} & \frac{d\varepsilon^{\left(1\right)}}{dt} & \tau^{\left(eq\right)}\;\\ \tau^{\left(vi\right)} & \tau^{\left(1\right)} & {\;\tau }_{\varepsilon^{\left(m\right)}}^{\left(1\right)} & \sigma \end{array}\right\|$$

It is easy to calculate the explicit form of the elements of this matrix as a function of the coefficients and, therefore, of the frequency of perturbation.

And finally:

(20) $$\sigma^{\left(s\right)}=\;\frac{1}{T}\left[\varepsilon_{o}^{2}G_{2R}\omega {cos}^{2}\omega t+\eta_{s}^{\left(1,1\right)}\tau^{(1)2}\right]$$

Writing this matrix for an isotropic viscoelastic medium means characterizing it in an almost univocal way. For further information on the formulas used, see Appendix A.

## 3. Results

The fit curves with the Zener model were extracted from the study of Sack et al. [11]; the results obtained allow for a comparison between young and old brains. For our considerations, we consider only the results up to 100 Hz even if the curve representation goes up to 500 Hz. Coefficients and thermodynamic functions referring to the old brain and young brain will be indicated with “o” and “y”, respectively.

Now, two new quantities are introduced by assuming that the CNS is made up of two parts with different anelastic characteristics: the glia and the neural tissue. Anelastic deformation ε^(1)^ is attributed to the glia indicated by $\varepsilon_{\left(g\right)}^{\left(1\right)}$ and to the neurons indicated with $\varepsilon_{\left(n\right)}^{\left(1\right)}$, such that:

(21) $$\varepsilon^{\left(1\right)}=\varepsilon_{\left(g\right)}^{\left(1\right)}+\varepsilon_{\left(n\right)}^{\left(1\right)}$$

**Figure 1:**
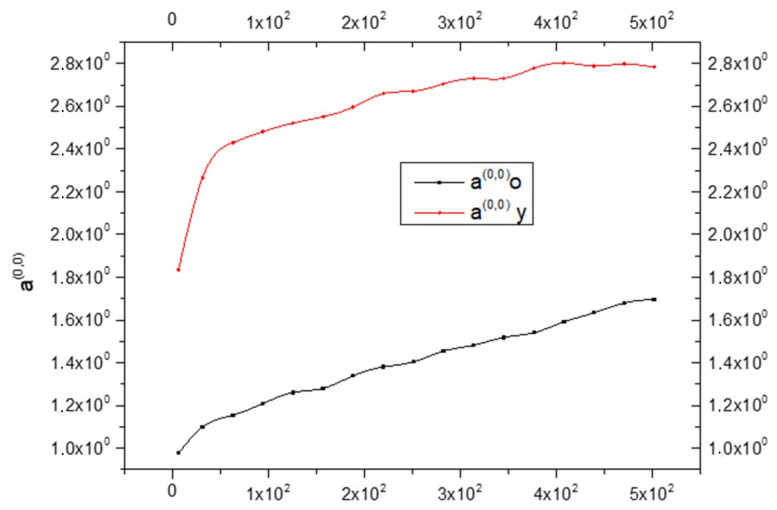
Comparison between the state coefficient a^(0,0)^ in young (black line) and old brains (red line) as a function of frequency ω; see Equation (16).

This will allow us to evaluate the prevalence of anelastic deformation in glia and neurons, assuming for them different states of inelasticity. Here, the individual components of the glia (astrocytes, microglia, and oligodendrocytes) will be neglected and considered as one body. The term “elastic deformation” will be used for ε^(0)^; this must not be misleading since its temporal derivative appears in the expression of entropy production. So, this term will be used only to distinguish it from the inelastic one, remembering that it is a non-elastic and therefore dissipative phenomenon. Figure 1 shows that it is always:

(22) $$a_{\left(y\right)}^{\left(0,0\right)}\;>\;a_{\left(o\right)}^{\left(0,0\right)}$$

**Figure 2:**
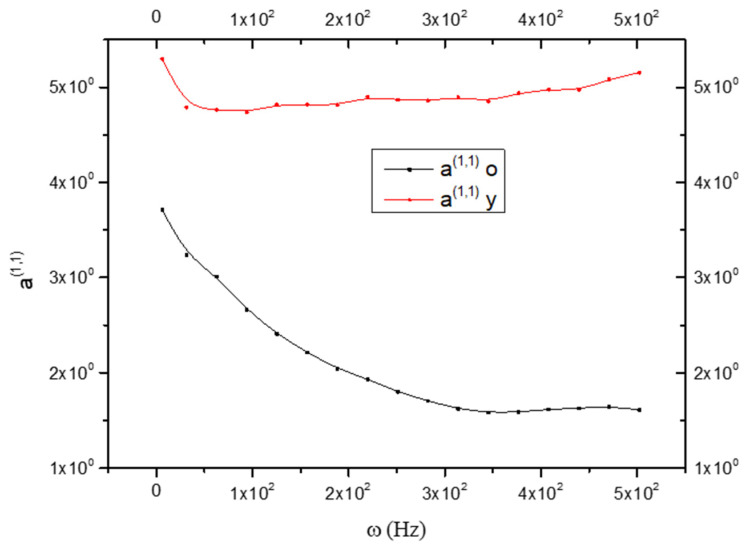
Comparison between the state coefficient a^(1,1)^ in old (black line) and young brains (red line) as a function of frequency ω; see Equation (17).

Since a^(0,0)^ is related to rigidity, therefore, it is possible to say that the CNS_(y)_ is more rigid than the CNS_(o)_. The same is shown in Figure 2, but while $a_{\left(y\right)}^{\left(0,0\right)}$ is an increasing function, $a_{\left(y\right)}^{\left(1,1\right)}$ is a decreasing function.

**Figure 3:**
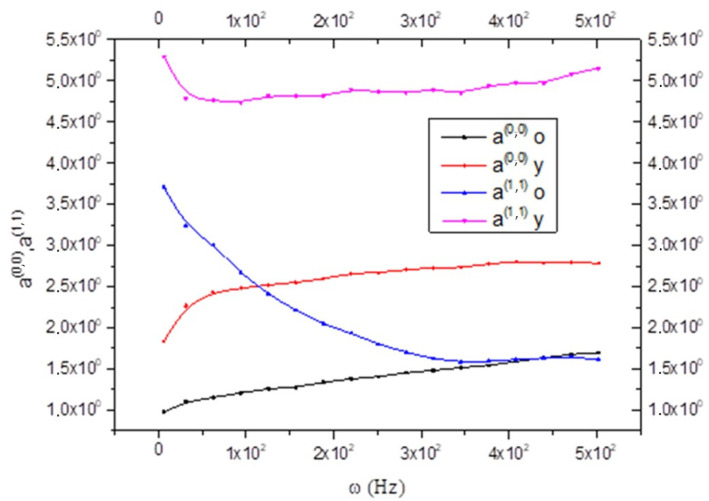
Comparison between the state coefficient a^(0,0)^ and a^(1,1)^ in old and young brains as a function of frequency ω; see Equations (16) and (17).

The different trend between the two coefficients is better seen by joining the two graphs together, as can be seen in Figure 3.

**Figure 4:**
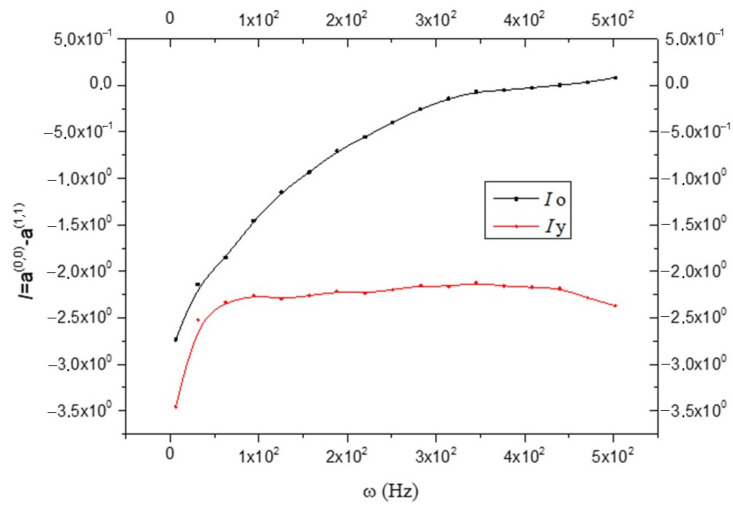
Comparison between the anelastic coefficient I in old (black line) and young brains (red line) as a function of frequency ω; see Equation (23).

Opposite behavior is shown in Figure 4, where the anelastic coefficient

(23) $$J=(a^{\left(0,0\right)}-a^{\left(1,1\right)})$$

is greater in the CNS_(o)_ than in the CNS_(y)_:

(24) $$I_{\left(o\right)}>I_{\left(y\right)}$$

**Figure 5:**
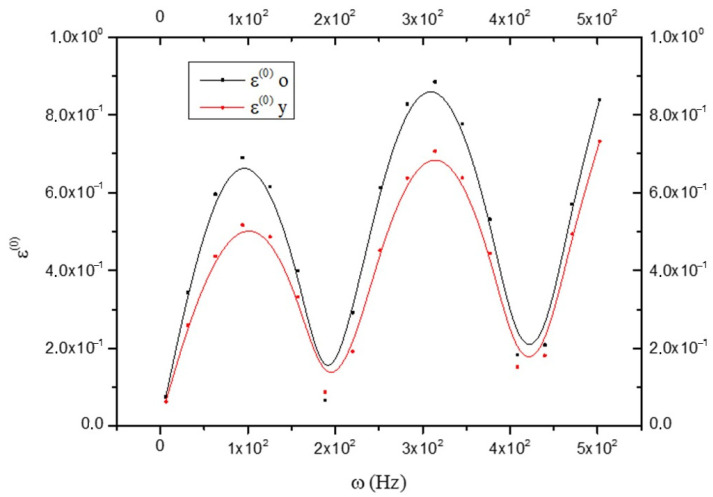
Comparison between the elastic strain ε^(o)^ in old (black line) and young brains (red line) as a function of frequency ω.

In this case, anelasticity is prevented to a greater extent than in the old brain. I_(y)_ remains practically constant at each frequency, which means that the anelastic characteristics do not change with the variation of ω; while I_(o)_ increases with ω (see Figure 4). In the expressions of ε^(0)^, ε^(1)^, and $\tau_{m}^{\left(1\right)}$, even if not specified, only absolute values will be considered. This is because the numerical value of the functions, and not the sign introduced by the trigonometric functions that appear in the expressions, are of interest. Furthermore, the phenomena studied are related to the displacements from equilibrium induced by the harmonic perturbation of shear, regardless of the direction in which it occurs (the medium is assumed to be isotropic). It is good to keep in mind that as the oscillation frequency increases, “smaller” elements and fewer large elements are affected. Looking at Figure 5, it results that:

(25) $$\varepsilon_{\left(o\right)}^{\left(0\right)}>\varepsilon_{\left(y\right)}^{\left(0\right)}$$

That is, the elastic part of the deformation is greater in the CNS_(o)_ than in the CNS_(y)_.

**Figure 6:**
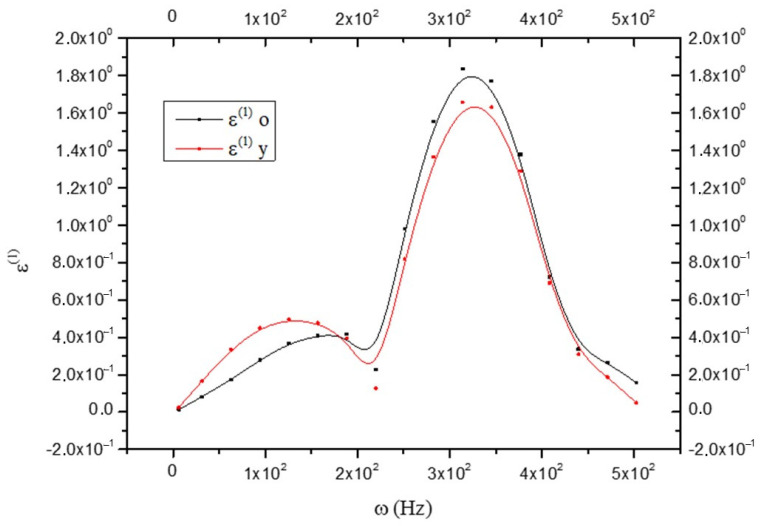
Comparison between the anelastic strain ε^(1)^ in old (black line) and young brains (red line) as a function of frequency ω.

This accords well with Equation (22), which shows a greater rigidity of the CNS_(y)_ compared to the CNS_(o)_. By observing Figure 6, it results that

(26) $$\varepsilon_{\left(y\right)}^{\left(1\right)}>\varepsilon_{\left(o\right)}^{\left(1\right)}$$

for (0<ω<180) Hz. That is, the anelastic part of the deformation is greater in the CNS_(y)_ but in a small range of frequencies, while, in accordance with Equation (24), it results that

(27) $$\varepsilon_{\left(o\right)}^{\left(1\right)}>\varepsilon_{\left(y\right)}^{\left(1\right)}$$

for ω>180 Hz (see Figure 6).

That is, our results indicate a minor rigidity of the CNS_(o)_ compared to the CNS_(y)_; this causes an anelasticity increase (see Figure 6). The anelastic coefficient J = (a^(0,0)^ − a^(1,1)^) is considered to be composed of a part related to the glia and one to the neurons, such that:

(28) $$J=J^{\left(g\right)}+J^{\left(n\right)}$$

where J^(g)^ is the part related to the glia and J^(n)^ is related to the neurons. From Equation (24), it results that:

(29) $$J_{\left(o\right)}^{\left(g\right)}+J_{\left(o\right)}^{\left(n\right)}\;>J_{\left(y\right)}^{\left(g\right)}+J_{\left(y\right)}^{\left(n\right)}$$

or:

(30) $$J_{\left(o\right)}^{\left(n\right)}{-J}_{\left(y\right)}^{\left(n\right)}\;>J_{\left(y\right)}^{\left(g\right)}-J_{\left(o\right)}^{\left(g\right)}$$

If the CNS_(o)_ loses neurons, it acquires a greater anelasticity, and therefore:

(31) $$J_{\left(o\right)}^{\left(n\right)}\;\text{>}J_{\left(y\right)}^{\left(n\right)}$$

and from Equation (31):

(32) $$J_{\left(o\right)}^{\left(g\right)}\;\text{>}J_{\left(y\right)}^{\left(g\right)}$$

That is, the anelastic component of the glia coefficient is greater in the CNS_(o)_ than in the CNS_(y)_. Recalling that the glia is composed of various elements but physiological aging is accompanied by degenerations of neurons and oligodendrocytes [23], for aging purposes, we only consider oligodendrocytes of the glia:

g = ol + x(33)

where “g” stands for glia, “ol” stands for oligodendrocytes, and “x” is the elements of the glia whose effects are neglected because they do not participate in aging. The Equation (32) is rewritten:

(34) $$J_{\left(o\right)}^{\left(ol+x\right)}\;\text{>}J_{\left(y\right)}^{\left(ol+x\right)}$$

Oligodendrocytes, due to their constitution, may not have cross effects with x; therefore, we can write:

(35) $$J^{\left(ol+x\right)}=J^{\left(ol\right)}+J^{\left(x\right)}$$

and Equation (34) becomes:

(36) $$J_{\left(o\right)}^{\left(ol\right)}+J_{\left(o\right)}^{\left(x\right)}\;>J_{\left(y\right)}^{\left(ol\right)}+J_{\left(y\right)}^{\left(x\right)}$$

**Figure 7:**
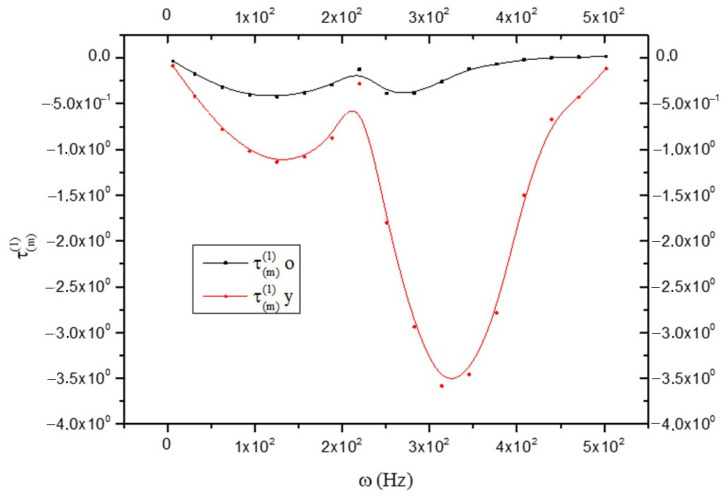
Comparison between the memory stress tensor in old (black line) and young brains (red line) as a function of frequency ω.

Since, as mentioned above, physiological aging is accompanied by the degeneration of oligodendrocytes and neurons, in Equation (36) it results that:

(37) $$J_{\left(o\right)}^{\left(x\right)}\;=J_{\left(y\right)}^{\left(x\right)}$$

and:

(38) $$J_{\left(o\right)}^{\left(ol\right)}\;>J_{\left(y\right)}^{\left(ol\right)}$$

the anelastic component of the coefficient J of “ol” is greater in the CNS_(o)_. It is important to underline that for the whole CNS, Equation (27) has been obtained (see Figure 4). This allows us to identify in the oligodendrocyte of the CNS_(o)_ the anelastic component that justifies Equation (24). Of course, if the CNS_(o)_ is more anelastic than the CNS_(y)_, as shown by Equation (32), it will “react” with greater stress; therefore, it will be as follows (remembering that J<0):

(39) $$\tau_{m(o)}^{\left(1\right)}>\tau_{m(y)}^{\left(1\right)}$$

according to Figure 4 and Figure 6 and Figure 7.

**Figure 8:**
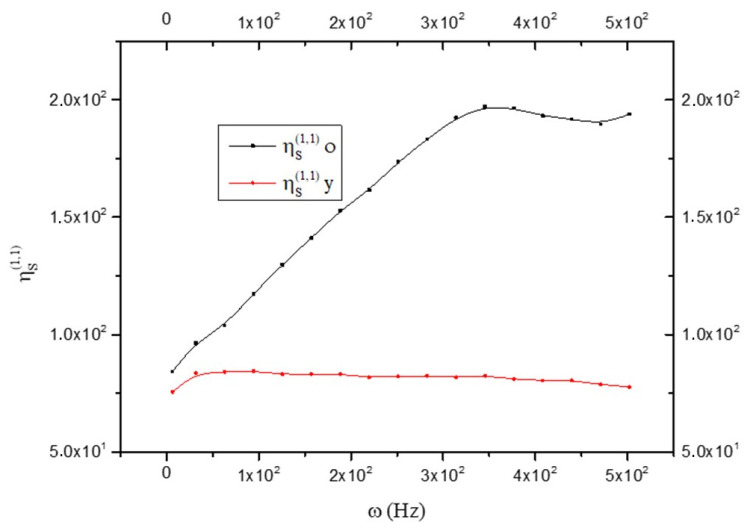
Comparison between the fluidity in old (black line) and young brains (red line) as a function of frequency ω; see Equation (18).

By observing Figure 8, we note that the following results:

$$\eta_{S(o)}^{\left(1,1\right)}>\eta_{S(y)}^{\left(1,1\right)}$$

**Figure 9:**
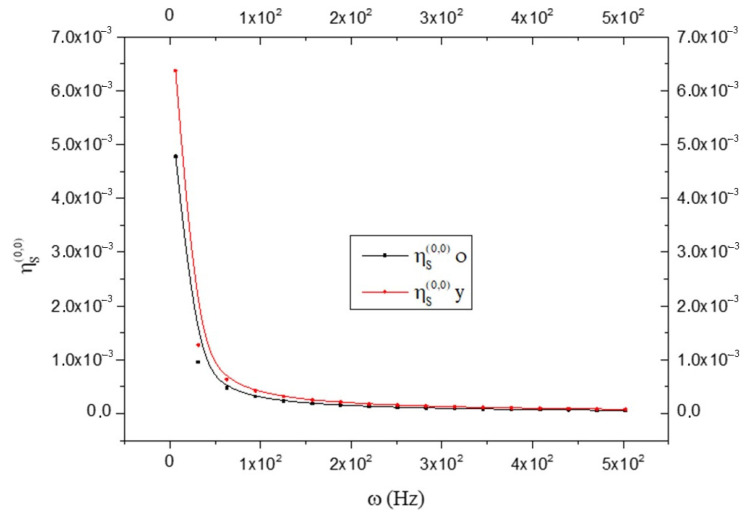
Comparison between the viscosity (expressed in Pascal x t) in old (black line) and young brains (red line) as a function of frequency ω; see Equation (19).

This means that the old brain is more fluid than the young brain. On the contrary, the viscosity is greater in the young brain than in the old one; i.e.:

$$\eta_{S(o)}^{\left(0,0\right)}<\eta_{S(y)}^{\left(0,0\right)}$$

as shown in Figure 9.

**Figure 10:**
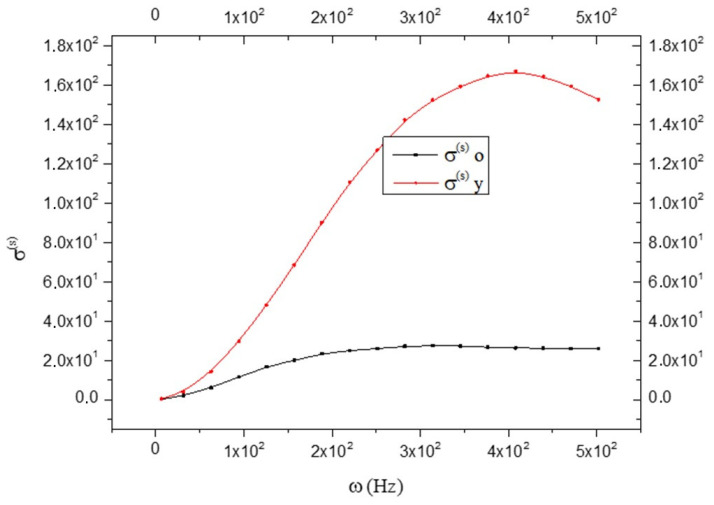
Comparison between the entropy production trend (expressed in Joules/Kelvin) in old (black line) and young brains (red line) as a function of frequency ω; see Equation (20).

Figure 10 shows the trend of entropy production. We note that it is clearly greater in the CNS_(y)_ than in the CNS_(o)_. This means that there is a greater “disorder” in the CNS_(y)_ in respect to the CNS_(o)._

## 4. Discussion

This study applies the non-equilibrium thermodynamic theory with internal variables to analyze the rheological properties of the brain, focusing on the in-depth analysis of the viscoelastic properties. It uses data derived from multifrequency magnetic resonance elastography measurements on the healthy human brains of 55 volunteers while not considering the difference based on gender, only age groups [11]. In detail, the application of four vibration frequencies in an acoustic range from 25 to 62.5 Hz and the use of the rheological spring model allowed for the determination of two parameters describing the solid–fluid behavior and microstructure of the brain tissue. From the results, it is evident that the CNS_(y)_ is more rigid than the CNS_(o)_. This is explained by remembering that the neural tissue is more rigid than the glia, and the CNS_(o)_ is characterized by neuron degeneration with partial myelin loss. Also, the minor rigidity of CNS_(o)_ compared to the CNS_(y)_ may be related to the loss of neurons with age, confirming what has been reported in the literature [23,24]; this causes an anelasticity increase, as is evident from Figure 4 and Figure 6.

Remembering that the anelastic coefficient J is due to a part related to the glia and one to the neurons since physiological aging is accompanied by the degeneration of oligodendrocytes and neurons, the anelastic component of the coefficient J of “ol” is greater in the CNS_(o)_ [25]. Then, we can identify in the oligodendrocyte of the CNS_(o)_ the anelastic component. Also, if CNS_(o)_ is more anelastic than CNS_(y)_, it will “react” with greater stress.

Analyzing the other studies’ results, it clearly emerges that the old brain is more fluid than the young brain; on the contrary, the viscosity is greater in the young brain than in the old one, as confirmed by other studies on the subject [26]. This can be justified by considering that in an old brain, the neuron degeneration causes a loss of “compactness”.

Furthermore, the entropy production is greater in the CNS_(y)_ than in the CNS_(o)_. Recalling that living systems constantly exchange matter and energy with the surrounding environment, for this reason, they can be considered complex dynamic systems far from a thermodynamic equilibrium condition [27]. Further, the degree of entropy generation in cells decreases with age, the generation of entropy is three times higher in infants than in the elderly [28], and the lower degree of entropy measured in the old human brain can be seen as a biological marker of age. In addition, according to Sohal’s theory, the age and metabolic degrees of organisms are inversely related [29]. In the brain, the oxidation of glucose for energy purposes is associated with the production of entropy. Therefore, the lower degree of entropy production monitored in the old brain compared to the young one inevitably indicates the lower metabolic activity of the neurons. Furthermore, changes in brain viscosity can affect the movement of molecules, including neurotransmitters, within the brain, disrupting normal neuronal function and signaling [30].

Aging can lead to a reduction in the number of synapses, impacting neuronal communication and cognitive function. Dendritic regression and the loss of these extensions are a common feature of aging and neurodegenerative diseases [31]. In this context, also based on data from the literature, the calculated statements $\;{\;\eta }_{S(o)}^{\left(1,1\right)}$ > $\eta_{S(y)}^{\left(1,1\right)}$ and $\eta_{S(o)}^{\left(0,0\right)}$ < $\eta_{S(y)}^{\left(0,0\right)}$ could be interpreted as the result of the dendritic regression associated with neuronal death, both potential consequences of an increased production of ROS due to reduced antioxidant defenses and/or an altered mitochondrial dysfunction in aging [32]. The slow but progressive altered mitochondrial functionality leads to an impaired Ca^2+^ handling [33,34,35]. A shift in Ca^2+^ regulation is strictly related to changes in cell excitability and synaptic plasticity, resulting in a functional lesion of the hippocampus [36,37]. Aging induces an increase in Ca^2+^ channel activity in the plasma membrane of brain neurons, leading to augmentation of cellular calcium content and resulting in alterations in bilayer fluidity relevant to the enzyme activity [38].

## 5. Conclusions

The novelty of this mathematical study is to provide a more detailed investigation about dissipative phenomena within the G_2_ calculated by Sack et al. [11]. The mathematical processing of what was measured by Ref. [11] has made it possible to distinguish two different dissipative phenomena expressed in G_2_; it could be said that this present study starting from a macroscopic phenomenon enables the researcher to observe microscopic phenomena that would otherwise not be analyzable. So, we could compare what has been processed as a sort of microscope that allows for a much more detailed analysis of the phenomena that occur in biological tissues—in this case, the CNS. The limitation of our study was to start from analyses already carried out in previous work [11]. If we had had more experimental data available, our mathematical elaboration could have been even more in-depth, but despite the limitations, we were able to highlight that aging reduces entropy production and increases anelasticity in brain tissue. In fact, our mathematical calculations have shown that:

- The CNS_(y)_ has more rigidity (Figure 1) and fewer anaelastic properties (Figure 4) than the CNS_(o)_ in the entire frequency range tested;
- Oligodendrocytes are the principal responsible for Equation (32), which is related to the anaelastic coefficient I;
- The CNS_(o)_ shows more fluidity and less viscosity than the CNS_(y)_ in the entire frequency range;
- The entropy production increases in the CNS_(y)_ compared to the CNS_(o)_, indicating more disorder in the young brain compared to the old brain.

In conclusion, starting from previous works on the topic [11,23,24,25,26,27], this study of the rheological properties of the central nervous system through a thermodynamic approach has led to new insights into the characteristics of the young and old brains, allowing for new knowledge of the phenomena involved. The available evidence, still mostly in the preclinical setting, indicates that brain viscoelasticity could become a valid diagnostic biomarker, in particular, through MRE. One of the most promising clinical applications concerns the estimation of the so-called “brain age gap” (the difference between biological brain age and chronological age), values that suggest “older” than expected mechanical properties could signal an increased risk of neurodegeneration or cerebrovascular disease. In this scenario, viscoelasticity is configured as an additional modality capable of enriching brain age models based on structural or functional MRI, representing the most concrete diagnostic perspective in the short term.

## Data Availability

The data that support the findings of this study are available from the corresponding author upon reasonable request.

## Abbreviations

The following abbreviations are used in this manuscript:

CNS	Central Nervous System
EEG	Electroencephalography
MEG	Magneto–encephalography
MRE	Magnetic resonance elastography
ROS	Reactive Oxygen Species
TMDB	Thermodynamic Matrix Dynamic Behavior
*Glossary*	
*s*	$\mathrm{entropy}\;s=s(u|{Ɛ}_{ik})$
*u*	internal energy
Ɛ_ik_	strain tensor
T	temperature
$\tau_{ik}^{\left(vi\right)}$	viscous stress tensor
$\tau_{ik}$	stress tensor that occurs in indefinite equations
*σ^(s)^*	entropy production
*t*	time
η	phenomenological coefficient
$\tau^{\left(1\right)}$	intensive variable
*f*	free energy
a^(0,0)^ and a^(1,1)^	State coefficients that have the dimension of a pressure.
ρ	mass density
*V *	velocity of the fluid element
d	relaxation time
G	G_1_ + iG_2_ dynamic module
G_1_	Storage Modulus
G_2_	Loss Modulus
$G_{2}^{\left(1\right)}$	inelastic “part” of the loss modulus G_2_
$G_{2}^{\left(1\right)}$	anelastic loss modulus
⍵	perturbation frequency
G_2R_	relaxed value of G_2_
$R_{o}^{\left(\tau \right)}$	internal resistance to deformation
$R_{1}^{\left(\varepsilon \right)}$	viscoelastic coefficient
σ	relaxation time
η^(0,0)^	dynamical coefficient = viscosity
η^(1,1)^	dynamical coefficient = fluidity
$\tau^{\left(vi\right)}$	viscous stress tensor
$\tau^{\left(eq\right)}$	equilibrium stress tensor

## Appendix A

In the theory, it is assumed that the specific entropy (which we indicate with s) has the following functional dependence [17]:

(A1) $$s=s\left(u\left|\varepsilon_{ik}\left|\varepsilon_{ik}^{\left(1\right)}\right.\right.\right)$$

from which it follows:

$$\left\{\begin{array}{c} \frac{1}{T}=\frac{\partial s\left(u\left|\varepsilon_{ik}\left|\varepsilon_{ik}^{\left(1\right)}\right.\right.\right)}{\partial u}\\ \tau_{ik}^{\left(eq\right)}=-T\rho \frac{\partial s\left(u\left|\varepsilon_{ik}\left|\varepsilon_{ik}^{\left(1\right)}\right.\right.\right)}{\varepsilon_{ik}}\\ \tau_{ik}^{\left(1\right)}=T\frac{\partial s\left(u\left|\varepsilon_{ik}\left|\varepsilon_{ik}^{\left(1\right)}\right.\right.\right)}{\partial \varepsilon_{ik}^{\left(1\right)}} \end{array}\right.$$

where it can be shown that by assuming that there exists a state *R* at the constant temperature *T*_0_, it results:

$$\tau_{ik}^{\left(eq\right)}\left(T_{o}\right)=\tau_{ik\left(0\right)}^{\left(eq\right)}=0$$

From Equation (A1), we obtain:

(A2) $$\frac{ds}{dt}=\frac{\partial s}{\partial U}\frac{dU}{dt}+\frac{\partial s}{\partial {Ɛ}_{ik}}\frac{d{Ɛ}_{ik}}{dt}+\frac{\partial s}{\partial {Ɛ}_{ik}^{\left(1\right)}}\frac{d{Ɛ}_{ik}^{\left(1\right)}}{dt}$$

Taking into account Equation (A1), it follows from (A2):

(A3) $$\frac{ds}{dt}=\frac{1}{T}\left[\frac{dU}{dt}-\tau_{ik}^{\left(eq\right)}\frac{d{Ɛ}_{ik}}{dt}+\tau_{ik}^{\left(1\right)}\frac{{dƐ}_{ik}^{\left(1\right)}}{dt}\right]$$

The first law of thermodynamic for unit of volume reads:

(A4) $$\frac{dU}{dt}=div{\underline{J}}^{\left(q\right)}+\tau_{ik}\frac{d{Ɛ}_{ik}}{dt}$$

where ${\underline{J}}^{\left(q\right)}$ is the heat flow.

By means of (A4), Equation (A3) can be written:

(A5) $$\frac{ds}{dt}=-\frac{1}{T}div{\underline{J}}^{\left(q\right)}+\frac{1}{T}\left(\tau_{ik}-\tau_{ik}^{\left(eq\right)}\right)\frac{d{Ɛ}_{ik}}{dt}+\frac{1}{T}\tau_{ik}^{\left(1\right)}\frac{d{Ɛ}_{ik}^{\left(1\right)}}{dt}$$

Taking into account Equation (4), the previous equation can be written:

(A6) $$\frac{ds}{dt}=-div\left(\frac{{\underline{J}}^{\left(q\right)}}{T}\right)+\sigma^{\left(s\right)}$$

where:

(A7) $$\sigma^{\left(s\right)}=\frac{1}{T}\left[-\frac{1}{T}{\underline{J}}^{\left(q\right)}gradT+\tau_{ik}^{\left(vi\right)}\frac{d{Ɛ}_{ik}}{dt}+\tau_{ik}^{\left(1\right)}\frac{d{Ɛ}_{ik}^{\left(1\right)}}{dt}\right]$$

Equation (A6) is the balance equation for the entropy, $\frac{1}{T}{\underline{J}}^{\left(q\right)}$, which is the entropy flow, and $\sigma^{\left(s\right)}$ is the entropy production.

Since we consider *T* = cost in the brain, Equation (A7) becomes:

(A8) $$\sigma^{\left(s\right)}=\frac{1}{T}\left[\tau_{ik}^{\left(vi\right)}\frac{d{Ɛ}_{ik}}{dt}+\tau_{ik}^{\left(1\right)}\frac{d{Ɛ}_{ik}^{\left(1\right)}}{dt}\right]$$

and taking into account Equation (3), we obtain:

(A9) $$\sigma^{\left(s\right)}=\frac{1}{T}\left[\tau_{ik}^{\left(vi\right)}\frac{d{Ɛ}_{ik}^{\left(0\right)}}{dt}+\left(\tau_{ik}^{\left(vi\right)}+\tau_{ik}^{\left(1\right)}\right)\frac{d{Ɛ}_{ik}^{\left(1\right)}}{dt}\right]$$

The free energy f is defined by [13]:

f=u−Ts

It can be shown that there exists the following functional dependence of *f*:

$$f=f\left(T\left|\varepsilon \left|\varepsilon^{\left(1\right)}\right.\right.\right)$$

This is the specific free energy, and it can be shown that the condition of isotropy and the linearity of the state equations are fulfilled if it is assumed that *f* is the sum of two functions *f*_1_ and *f*_2_ [13,15]:

(A10) $$f=f_{1}+f_{2}$$

where:

(A11) $$f_{1}=f_{1}\left(T\left|\varepsilon_{ik}\right.\right)$$

(A12) $$f_{2}=\frac{1}{2}\left[a^{\left(0,0\right)}\varepsilon \cdot \left(\varepsilon -2\varepsilon^{\left(1\right)}\right)+a^{\left(1,1\right)}\varepsilon^{{\left(1\right)}^{2}}\right]$$

where *a*^(0,0)^ and *a*^(1,1)^ are state coefficients (assumed constant) that have the dimension of a pressure.

We can obtain the following state equations [18]:

(A13) $$\tau^{\left(eq\right)}=a^{\left(0,0\right)}\left(\varepsilon -\varepsilon^{\left(1\right)}\right)=a^{\left(0,0\right)}\varepsilon^{\left(0\right)}$$

(A14) $$\tau^{\left(1\right)}=a^{\left(0,0\right)}\varepsilon -a^{\left(1,1\right)}\varepsilon^{\left(1\right)}$$

where, together with Equations (7) and (8), we neglect the cross effects that may occur between them:

(A15) $$\tau^{\left(vi\right)}=\eta_{s}^{\left(0,0\right)}\frac{d\varepsilon }{dt}$$

(A16) $$\frac{d\varepsilon^{\left(1\right)}}{dt}=\eta_{s}^{\left(1,1\right)}\tau^{\left(1\right)}$$

These will be the equations that allow us to introduce the relaxation equation.

The hypothesis that the tissue we are studying is an incompressible fluid is expressed by a fluid’s element, dτ, by the mathematical relation $\frac{d}{dt}\left(d\tau \right)=0$; so, from the well-known cinematic relation $\frac{d}{dt}\left(d\tau \right)=div\underline{V}d\tau$, it follows $div\underline{V}=0$. This last equation and the equation for the conservation of mass $\frac{d\rho }{dt}+\rho div\underline{V}=0$ lead us to assert that:

(A17) $$\frac{d\rho }{dt}=\frac{\partial \rho }{\partial t}+\underline{V}\cdot grad\rho =0$$

where $\underline{V}$ is the velocity of the fluid element. If the fluid is not homogenous at the initial instant, its initial density *ρ*_0_ varies with Lagrangian coordinates (*b*_1_,*b*_2_,*b*_3_), so as to have

$$\rho_{0}=\rho_{0}\left(b_{1},b_{2},b_{3}\right).$$

By substituting the following relation (which is valid during the motion) $b_{i}=b_{i}\left(t,x_{1},x_{2},x_{3}\right)$ in the last equation, one has:

$$\rho_{0}\left(b_{1},b_{2},b_{3}\right)=\rho_{0}\left[b_{i}\left(t,x_{i}\right)\right]=\rho \left(t,x_{1},x_{2},x_{3}\right)$$

This relation satisfies Equation (A17) and proves the assertion.

Thus, we assume that the mass density *ρ* is constant [8,9,10]. It is seen from Equation (14) that a sudden change in ε^(1)^ is impossible, while from Equation (A15), it follows that a sudden change in ε^(0)^ is possible.

It can be shown that it is possible to eliminate the internal fields and the two fields $\varepsilon^{\left(0\right)}$ and $\varepsilon^{\left(1\right)}$ from the Equations (A13)–(A16), so as to obtain the so called relaxation equation mentioned in the introduction [12,13,14,15,16,17,18,19]:

(A18) $$R_{0}^{\left(\tau \right)}\tau +\frac{d\tau }{dt}=R_{0}^{\left(\varepsilon \right)}\varepsilon +R_{1}^{\left(\varepsilon \right)}\frac{d\varepsilon }{dt}+R_{2}^{\left(\varepsilon \right)}\frac{d^{2}\varepsilon }{dt^{2}}$$

where:

(A19) $$R_{o}^{\left(\tau \right)}=a^{\left(1,1\right)}\eta^{\left(1,1\right)}=\frac{1}{\sigma }\;[R_{o}^{\left(\tau \right)}]=\frac{1}{t}$$

(A20) $$R_{o}^{\left(\varepsilon \right)}=a^{\left(0,0\right)}(a^{\left(1,1\right)}-a^{\left(0,0\right)})\eta_{s}^{\left(1,1\right)}[R_{o}^{\left(\varepsilon \right)}]={\mathrm{mL}}^{-1}t^{-3}$$

(A21)
$$R_{1}^{\left(\varepsilon \right)}=a^{\left(0,0\right)}+a^{\left(1,1\right)}\eta_{s}^{\left(1,1\right)}\eta_{s}^{\left(0,0\right)}[R_{1}^{\left(\varepsilon \right)}]={\mathrm{mL}}^{-1}t^{-2}$$

(A22)
$$R_{2}^{\left(\varepsilon \right)}=\eta_{s}^{\left(0,0\right)}\;[R_{2}^{\left(\varepsilon \right)}]={\mathrm{mL}}^{-1}t^{-1}$$

Here, σ is the relaxation time, and the index “s” that appears in $\eta^{\left(0,0\right)}$ and $\eta^{\left(1,1\right)}$ indicates “shear”.

It is easy to calculate the explicit form of the elements of the TMDB as functions of the aforementioned coefficients and, therefore, of the frequency of perturbation. They are as follows:

(A23) $$\tau^{\left(vi\right)}=\eta_{s}^{\left(0,0\right)}\;\varepsilon_{o}\omega cos\omega t$$

(A24) $$\tau^{\left(eq\right)}=\varepsilon_{o}G_{1}sen\omega t+\varepsilon_{o}G_{2}cos\omega t-\varepsilon_{o}\eta_{s}^{\left(0,0\right)}\omega cos\omega t$$

(A25) $$\varepsilon^{\left(0\right)}=\frac{\tau^{\left(eq\right)}}{a^{\left(0,0\right)}}=\varepsilon_{o}\frac{\left[G_{1}sen\omega t+\left(G_{2}^{\left(1\right)}\right)cos\omega t\right]}{a^{\left(0,0\right)}}$$

(A26) $$\varepsilon^{\left(1\right)}=\varepsilon_{o}\left(1-\frac{G_{1}}{a^{\left(0,0\right)}}\right)\mathrm{sen}\omega t-\varepsilon_{o}\frac{\left(G_{2}^{\left(1\right)}\right)}{a^{\left(0,0\right)}}cos\omega t$$

(A27) $$\tau^{\left(1\right)}=\frac{1}{\eta_{s}^{\left(1,1\right)}}[\varepsilon_{o}\omega \left(1-\frac{G_{1}}{a^{\left(0,0\right)}}\right)cos\omega t+\varepsilon_{o}\omega \frac{\left(G_{2}^{\left(1\right)}\right)}{a^{\left(0,0\right)}}sen\omega t]$$

(A28) $$\frac{d\varepsilon^{\left(1\right)}}{dt}=\varepsilon_{o}\omega \left(1-\frac{G_{1}}{a^{\left(0,0\right)}}\right)cos\omega t+\varepsilon_{o}\omega \frac{\left(G_{2}^{\left(1\right)}\right)}{a^{\left(0,0\right)}}sen\omega t$$

(A29) $$\tau_{m}^{\left(1\right)}=\left(a^{\left(0,0\right)}-a^{\left(1,1\right)}\right)\left[\varepsilon_{o}\left(1-\frac{G_{1}}{a^{\left(0,0\right)}}\right)sen\omega t-\varepsilon_{o}\frac{\left(G_{2}^{\left(1\right)}\right)}{a^{\left(0,0\right)}}cos\omega t\right]$$

And finally, from Equation (A10):

(A30) $$\sigma^{\left(s\right)}=\frac{1}{T}\left\{G_{2R}\varepsilon_{o}cos\omega t\varepsilon_{o}\omega cos\omega t+\tau^{\left(1\right)}\frac{d\varepsilon^{\left(1\right)}}{dt}\right\}=\frac{1}{T}\left[\varepsilon_{o}^{2}G_{2R}\omega {cos}^{2}\omega t+\eta_{s}^{\left(1,1\right)}\tau^{(1)2}\right]$$

that is:

(A31) $$\sigma^{\left(s\right)}=\frac{1}{T}\left[\varepsilon_{o}^{2}G_{2R}\omega {cos}^{2}\omega t+\eta_{s}^{\left(1,1\right)}\tau^{(1)2}\right]$$

Writing this matrix for an isotropic viscoelastic medium means characterizing it in an almost univocal way.
